# Supplementary material for: Effect of Delta and Omicron Mutations on the RBD-SD1 Domain of the Spike Protein in SARS-CoV-2 and the Omicron Mutations on RBD-ACE2 Interface Complex
Source: Int J Mol Sci. 2022 Sep 3;23(17):10091. doi: 10.3390/ijms231710091 (PMC9456519; doi:10.3390/ijms231710091)
Supplement: Supplementary file 1 [file ijms-23-10091-s001.zip › ijms-1866336-supplementary.pdf]

Supplementary Materials for

Effect of Delta and Omicron Mutations on the RBD-SD1 Domain of the Spike Protein in SARS-CoV-2 and the Omicron Mutations on RBD-ACE2 Interface Complex

Wai-Yim Ching [1,*], Puja Adhikari [1], Bahaa Jawad [1,2] and Rudolf Podgornik [3,4,5]

1 Department of Physics and Astronomy, University of Missouri-Kansas City, Kansas City, MO 64110, USA
2 Department of Applied Sciences, University of Technology, Baghdad 10066, Iraq
3 School of Physical Sciences and Kavli Institute of Theoretical Science, University of Chinese Academy of Sciences, Beijing 100049, China
4 CAS Key Laboratory of Soft Matter Physics, Institute of Physics, Chinese Academy of Sciences, Beijing 100090, China
5 Wenzhou Institute, University of Chinese Academy of Sciences, Wenzhou 325000, China
* Correspondence: chingw@umkc.edu

Content:

S1 Computational Methods

(a) VASP

One of the key feature in our computational strategy is to combine the two well established packages based on density functional theory (DFT): The Vienna *ab initio* Simulations package (VASP) [1] and the orthogonalized linear combination of atomic orbital OLCAO) method [2]. This strategy is pivotal for successful application to large and complex materials since more than 20 years ago. In recent years, it has been further demonstrated to be highly effective by using supercells containing large number of atoms. They include but not limited to complex crystals and disordered non-crystalline materials [3-9], biomolecules [10-25], proteins [14, 17-25], glasses [26-30] and different organic [31, 32], inorganic[11, 13, 33] and metallic systems [5, 34-36].

The initial structure for RBD-SD1 domain in S-protein is obtained from the selected source in protein data bank (PDB) with appropriate modification such as addition of missing hydrogen (H) atoms. The initial structure with several thousands of atoms is then placed in a large supercell with periodic boundary conditions. The supercell is sufficiently large to ensure no artificial interaction occurs between the large biomolecule and its periodic image. The model is then fully optimized to high precision using VASP [1], which is known for its efficiency in structural optimization. We adopt the usual projector augmented wave (PAW) method with Perdew-Burke-Ernzerhof (PBE) exchange correlation functional [37] within the generalized gradient approximation (GGA). This selection is one of the several options that balance the accuracy needed and the computational resources available. Detailed tests suggest that the use of the following input parameters to VASP for large biomolecular systems is more than sufficient: (1) energy cut-off at 500 eV; (2) electronic convergence of 10^{-4} eV for each step; (3) force convergence for ionic steps at -10^{-2} eV/Å; (4) a single k-point sampling at the center of the supercell (Γ). The final relaxed structure of the supercell models has achieved the accuracy of difference in total energy is less than -0.330 eV or -0.000108 eV per atom. This optimized structure is used as the input data for OLCAO calculation.

(b) OLCAO

The OLCAO package also based on DFT was developed by our group at the University of Missouri-Kansas City [2]. It is particularly effective for the calculation of electronic structure and interatomic interactions of large biomolecule systems few other DFT-based methods can match. In contrast to VASP, atomic orbitals are used for basis expansion in conjunction with in orthogonalization to the core orbitals protocol that enable us to diagonalize the huge matrix with a single step to obtain all the energy eigen values and wave functions of the Kohn-Sham equation [38]. Two fundamental quantities from the *ab initio* wave functions from OLCAO are most important: the effective charge (Q^*) on each atom and the bond order (BO) value $\rho_{\alpha\beta}$ between any pair atoms α and β in the supercell defined in Eq. (S1) and (S2) below.

$$Q_\alpha^* = \sum_i \sum_{m,occ} \sum_{j,\beta} C_{i\alpha}^{*m} C_{j\beta}^{m} S_{i\alpha,j\beta} \tag{S1}$$

$$\rho_{\alpha\beta} = \sum_{m,occ} \sum_{i,j} C_{i\alpha}^{*m} C_{j\beta}^{m} S_{i\alpha,j\beta} \tag{S2}$$

In equations (1) and (2), $S_{i\alpha,j\beta}$ are the overlap integrals between the ith orbital in the αth atom and the jth orbital in the βth atom. $C_{j\beta}^{m}$ are the eigenvector coefficients of the m^{th}occupied molecular orbital. We can the so-called partial charge (PC) from Q_α^* , which is the deviation of Q_α^* from the neutral atomic charge Q_α^0 on the same atom ($\Delta Q_\alpha = Q_\alpha^0 - Q_\alpha^*$). The BO represents the strength of the bond between two atoms in the unit of electrons (e^-). BO usually scales with the bond length (BL) or the distance of separation between atoms α and β, depending also on the local atomic configuration of the vicinal atoms. It should be explicitly pointed out that PC and BO in Eq. (1) and (2) are fundamentally different form other simulation methods which are fixed parameters such as in the force field specification in molecular dynamic (MD) simulation. Another point to emphasize is that Q* and BO are basis-dependent since they are based on the Mulliken scheme [39, 40] using localized atomic orbitals. We use the minimal basis in most of our calculations for large biomolecular systems. Another key important point is that OLCAO is a one-point calculation to obtain all BO values for all atomic pairs to characterize the internal cohesion of the system under study, rather than the traditional total energy or enthalpy calculation (two-point or even many-point calculations) which is used to describe the strength of binding between biomolecular systems. The sum of all BO values within a structural component such as in a protein and its subdomains gives the total bond order (TBO), which accurately describes the internal cohesion critical to analyzing the AA-AA network for large complex biomolecules.

(c) Analysis of Amino acid - amino acid bond pair unit (AABPU)

In complex biomolecules we need to extend the concept of the bond order (BO) values for a *pair of atoms* to interaction between a *pair of amino acids* (AAs). We refer to this generalized quantifier of molecular interactions the *amino acid-amino acid bond pair* (AABP) as first described in ref. [17]

$$AABP(u,v) = \sum_{\alpha\epsilon u} \sum_{\beta\epsilon v} \rho_{\alpha i,\beta j} \tag{S3}$$

In Eq. (3), the summations are over all atoms α in $AA\ u$ and all atoms β in $AA\ v$. AABP considers all possible bonding between two AAs including both covalent and hydrogen bonding (HB). AABP value is a single parameter proxy that quantifies the interaction between two AAs. The stronger the interaction, the higher will be the AABP value and vice versa irrespective of the nature and composition of the 20 canonical AAs. The specific structural unit that contains the relevant AAs is coined as AABPU. AABP value in each AABPU can be further resolved into different components, nearest neighbor (NN) in the amino acid sequence (NN-AAPB) and non-local (NL-AAPB) parts. It should be emphasized that the AABP does not involve the "BL" used for the description of interacting atoms since the distance of separation between two AAs is impossible to quantify precisely even people have been tried by using distance of separation between specific "C" atom in the AAs. AABP values are calculated from quantum mechanical wave functions of the entire biomolecular unit and thus represent a collective structural parameter, including the effects of all

atomic pairs involved. AABPU is a novel concept to measure of molecular interactions in biomolecules, that contains the nearest-neighbor or local interactions of AAs that are vicinal along the sequence and in the 3D folding space, as well as the off-diagonal or non-local interaction between AAs that are not vicinal in the sequence space but are interacting in 3D folding space. Clearly, this is a giant step forward in in the theory of biomolecular interaction.

(d) Graphical Illustration

The methods used for graphical illustrations are briefly outlined below: **Figure 1 (a)** is prepared using the PowerPoint whereas **Figure 1 (b)**, **(c)**, **(d)** and **Figures 2**, **3,** and **13** are prepared using Chimera [41]. All other figures (**Figures 4** to **Figure 12)** are prepared using Origin-version 8 software. The graphical illustration of AABPU in **Figure 3** and **Figure S1** entails the plots of a 3-dimensional (3D) structure of a collection of AAs on a 2D plane in atomic scale. This is a complex and time-consuming task. We proceed it as follows: Firstly, the data are from optimized structure using VASP followed by OLCAO calculations. The numerical data for the bonding between every pair of bonds are extracted are listed in a large table. These bonds are then analyzed in different groups such as bond types, (covalent or hydrogen bond), bonds formed by specific amino acids, mutated or unmutated (WT) etc. All amino acids involved in bonding with the mutated amino acids are considered. We then prepare the plots for the complicated 2D plane figure via Chimera [41].

S2 Partial density of states (PDOS) for WT, DV and OV in RBD-SD1

In **Figure S3**, we display the 18 PDOS following the order in **Table 1** with WT and DV or OV in the same figure. To simplify the discussion, the structural unit used in PDOS are the central amino acid for each model listed in **Table 1**. The following interesting observations are noted

A. PDOS for all panels in **Figure S3** are very close between WT, but the mutated types show many differences. This demonstrates the penetrating details can be revealed in PDOS.
B. The peak positions in PDOS are mostly similar and aligned, a fact consistent with all biological molecules consists of AAs.
C. The PDOS figures in **Figure S3** are for each main AA in **Table 1**. They should not be compared or correlated with AABPU since the later consists of additional NN, Nonlocal amino acids with contributions from HBs.
Still, for a single AA in the AABPU, some interesting observations can be identified. We will comment on each of the 18 AAs.
D. The comparative study is based on following observations: increase or decrease in the area under the curve which is the number of energy states it contains.
E. There are roughly two regions in the VB, the first major peak between 0 to -4 eV and the second dominating group of states below -4 eV.
F. In the unoccupied CB regions, they can also be roughly divided into a lower peak below 5 eV and all other states above it. They are the antibonding images of the states in the VB groups.

We succinctly comment on each PDOS figure by comparing the observed features in different regions before and after mutation.
1. DV L452: In both the VB and CB, the areas under the PDOS curves are increased after mutation.
2. DV K478: In the VB, both areas under the curves are increased after mutation except the first peak remain similar. The features in the CB are similar as the mirror image of the VB.
3. OV D339: In both the VB and CB, the areas under the PDOS curves are significantly increased after mutation.
4. OV L371: In both the VB and CB, the areas under the PDOS curves are increased after mutation except the trend is reversed in the first peak compared to the second one. The increase is larger in the second peak in CB than in the VB.

5. OV P373: The feature in both VB and CB are similar to OV L371 except the change after mutation is by a lesser amount.
6. OV F375: The feature in both VB and CB are similar to OV P373 except the change after mutation is by a larger amount.
7. OV N417: This figure is the first one in which the area under the curve in both VB and CB are decreased by a fairly large amount after mutation, especially in the second peak in the CB.
8. OV K440: The feature in both VB and CB are similar to OV P373 except the change after mutation is by a larger amount in the second peak of the CB.
9. OV S446: Both areas under the curve in VB and CB increased after mutation similar to K440.
10. OV N477: This site is very similar to OV N417 in almost all aspects. This is the second case where mutation resulted in the areas under the curve.
11. OV K478: This site is very similar to OV K440 in almost all aspects.
12. OV A484: This site is very similar to OV N477 in almost all aspects. This is the third case where mutation reduces the areas under the curve
13. OV R493: This site is very similar to OV K478 in almost all aspects.
14. OV S496: This site is almost identical to OV N477 in shapes of the curve but with very puzzling difference. Mutation in increases the area under the curves whereas OV N477 is opposite.
15. OV R498: This site is very similar to OV R493 in almost all aspects.
16. OV Y501: This site is again similar to OV R493 as well except the increase in areas under curves after mutation is slightly larger.
17. OV H505: This site is very similar to OV A484 in almost all aspects. This is the last example where mutation actually decreases the areas under the curves.
18. OV K547: This site is very similar to OV K493 in almost all aspects. We emphasize that this is the only site in the SD1 portion of the structural model, not the RBD.
19. The two DV sites have very similar PDOS spectra. Mutation increases the areas under the curves.
20. Out of 16 OV sites, 4 of them have mutation reduces the areas under the curve (25%). OV sites also have more variations among them such as difference between VB and CB regions. These observations all point to the complexity of Omicron variant in addition to large number of mutations.

Supplementary Figures:

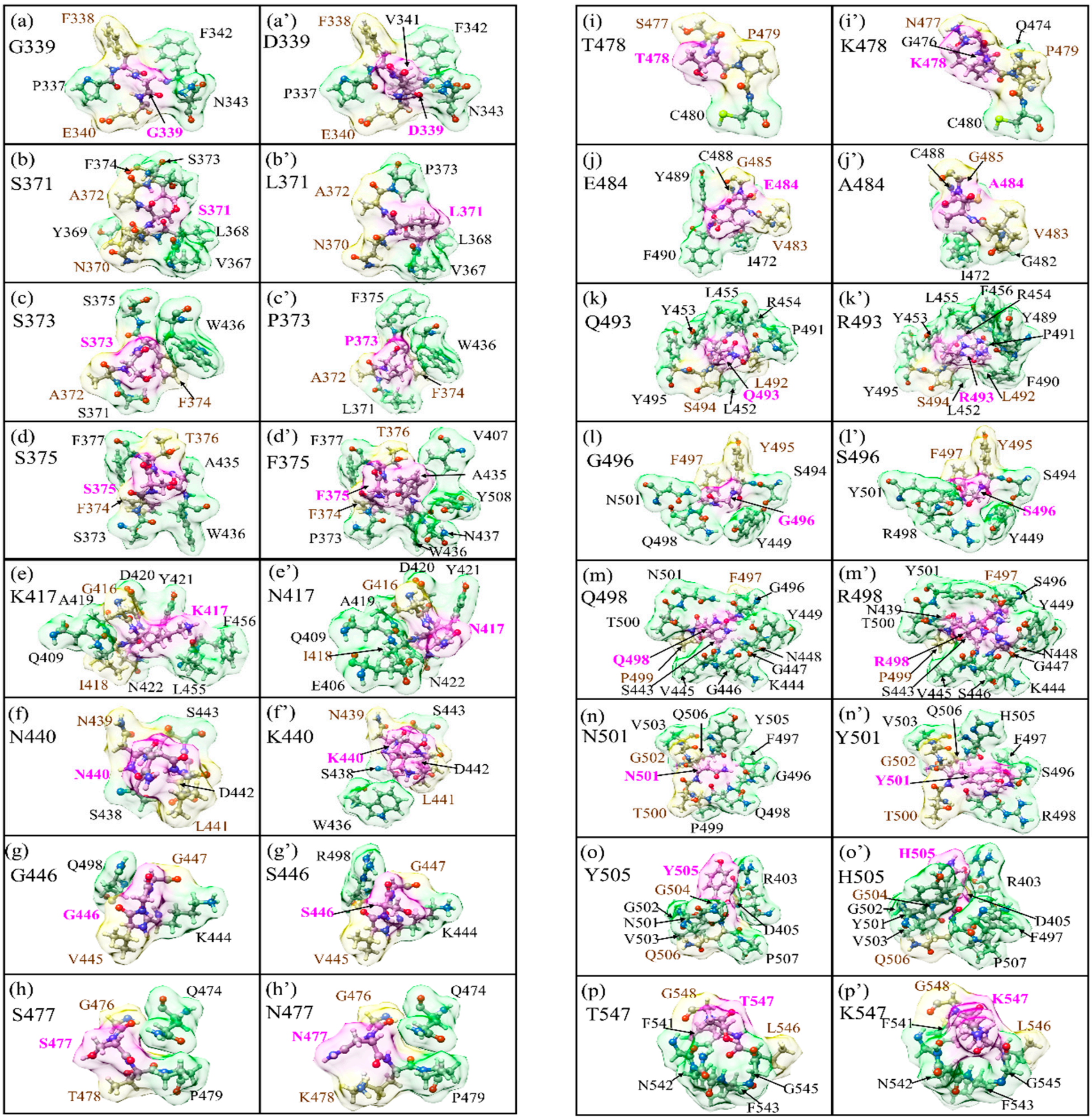

Figure S1. Details of the shape change of AABPU of the sixteen mutation sites in RBD-SD1: (a) G339, (b) S371, (c) S373, (d) S375, (e) K417, (f) N440, (g) G446, (h) S477, (i) T478, (j) E484, (k) Q493, (l) G496, (m) Q498, (n) N501, (o) Y505, and (p) T547 for the WT. (a') D339, (b') L371, (c') P373, (d') F375, (e') N417, (f') K440, (g') S446, (h') N477, (i') K478, (j') A484, (k') R493, (l') S496, (m') R498, (n') Y501, (o') H505, and (p') K547 for the OV. The surface of mutated sites is shown in magenta, surface of NN and NL are shown in yellow and green respectively. All NN and NL AAs are marked near to their surface in brown and black respectively.

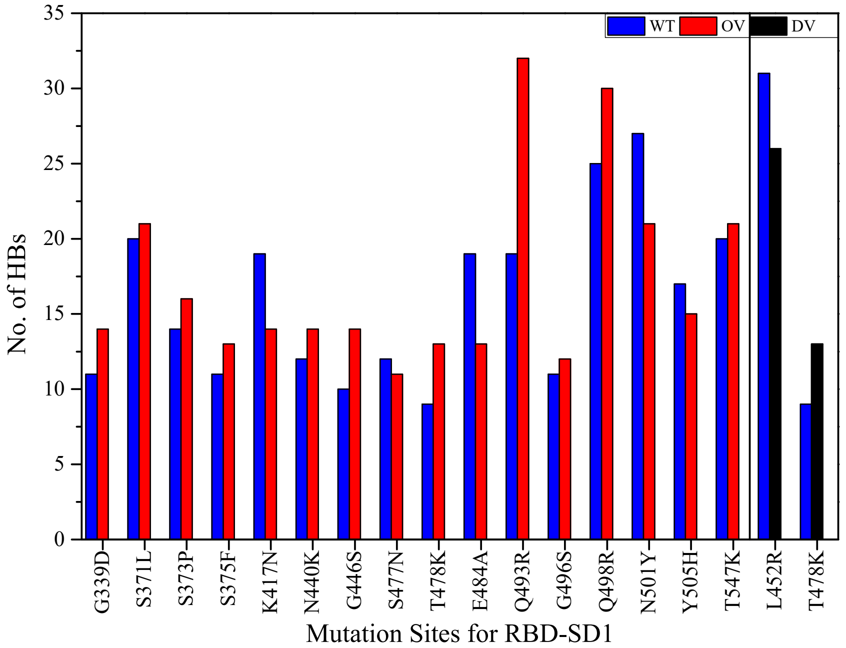

Figure S2. Number of hydrogen bonds in 18 mutation sites of RBD-SD1 including WT, DV, and OV.

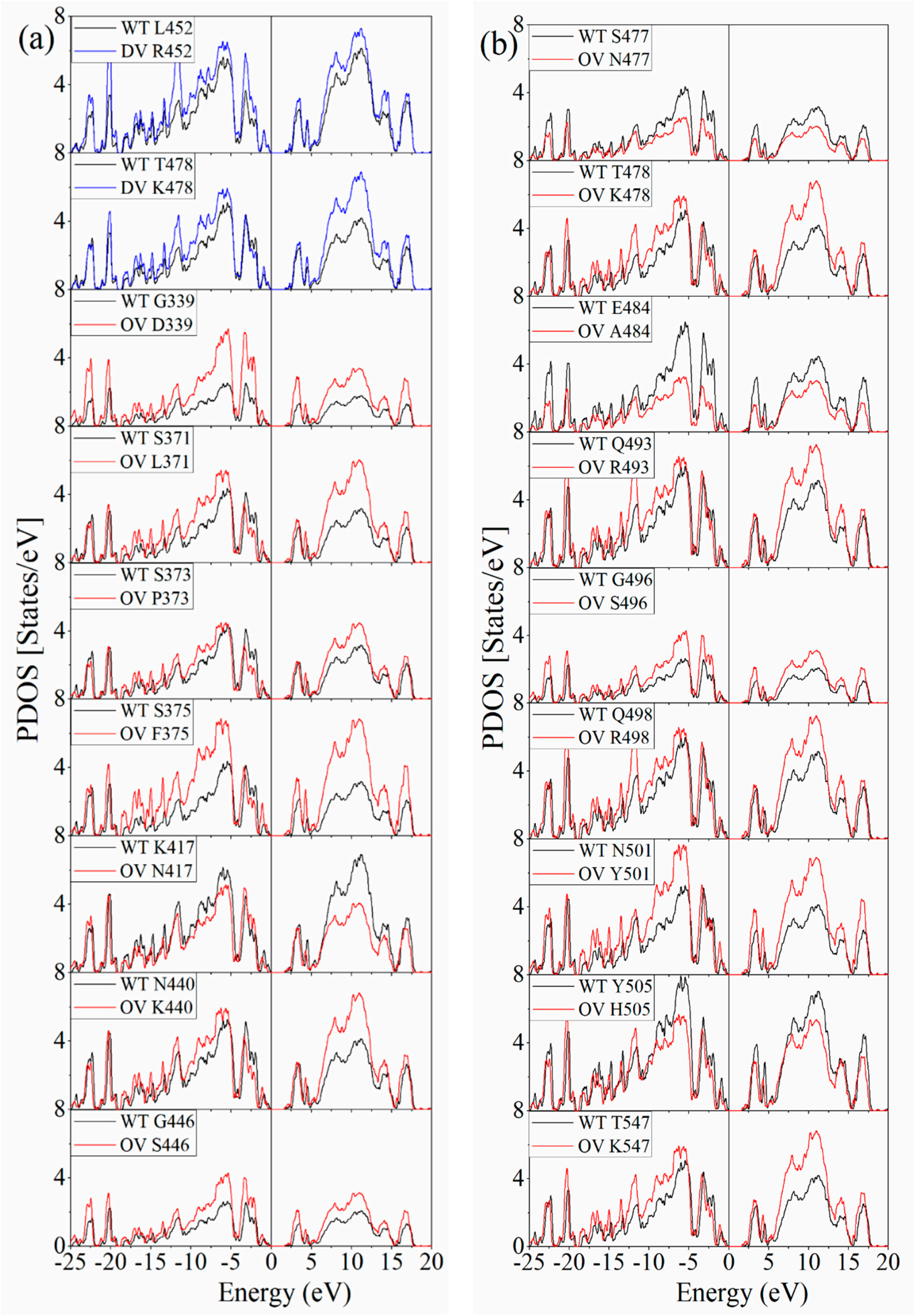

Figure S3. Comparison of PDOS per each amino acid for the 18 mutations (16 for OV, 2 for DV) with the WT. Black: WT, blue: DV, red: OV. For easy contrast, each panel for the listed AA site has two PDOS curves, WT and mutated one.

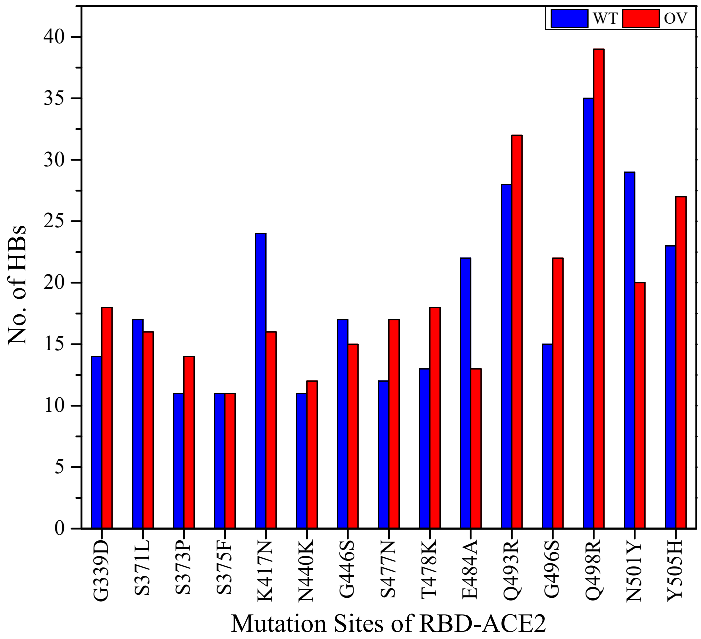

Figure S4. Number of hydrogen bonds in 15 mutation sites of RBD-ACE2 including WT and OV.

Supplementary Tables:

Table S1: Number of HBs and total bond order (TBO) in RBD-SD1 for WT, DV, and OV shown in **Figure 6**. The last column is the total data points for HB.

**HBs**		**O…H**	**N…H**	**Total**
**WT**	Count (BL<2.0Å)	133	4	
	Count (BL>2.0Å)	2391	1493	
	Count (Total)	2524	1497	4021
	TBO <2.0Å	6.2467	0.1819	
	TBO >2.0Å	7.7355	3.0292	
	TBO (Total)	13.9822	3.2111	17.1933
**DV**	Count (BL<2.0Å)	133	4	
	Count (BL>2.0Å)	2384	1516	
	Count (Total)	2517	1520	4037
	TBO <2.0Å	6.3117	0.1807	
	TBO >2.0Å	7.6313	3.1029	
	TBO (Total)	13.943	3.2836	17.2266
**OV**	Count (BL<2.0Å)	146	5	
	Count (BL>2.0Å)	2384	1549	
	Count (Total)	2530	1554	4084
	TBO <2.0Å	6.7742	0.3539	
	TBO >2.0Å	7.4242	3.2033	
	TBO (Total)	14.1984	3.5572	17.7556

**Table S2**: PC^{AA} for ACE2 AAs of WT model. Color coded according to **Figure 13**. The residues in ACE2: 19-88 and 319-365.							
**AAs**	**PC^{AA}**	**AAs**	**PC^{AA}**	**AAs**	**PC^{AA}**	**AAs**	**PC^{AA}**
S19	0.644	W48	0.025	S77	-0.015	P336	0.047
T20	-0.118	N49	0.045	T78	-0.030	G337	-0.027
I21	0.023	Y50	0.030	L79	-0.060	N338	0.120
E22	-0.393	N51	-0.071	A80	0.002	V339	0.057
E23	-0.549	T52	-0.067	Q81	-0.016	Q340	0.017
Q24	0.061	N53	0.036	M82	-0.108	K341	0.657
A25	0.002	I54	0.017	Y83	-0.061	A342	0.042
K26	0.512	T55	-0.157	P84	0.057	V343	-0.089
T27	-0.070	E56	-0.815	L85	0.135	C344	0.123
F28	0.002	E57	-0.780	Q86	-0.105	H345	-0.132
L29	0.040	N58	-0.034	E87	-1.010	P346	0.144
D30	-0.446	V59	0.010	I88	-0.998	T347	-0.101
K31	0.573	Q60	-0.045	G319	1.031	A348	0.048
F32	-0.081	N61	0.053	L320	-0.184	W349	1.898
N33	-0.016	M62	-0.034	P321	0.162	D350	-0.814
H34	0.078	N63	-0.016	N322	-0.034	L351	-0.089
E35	-0.723	N64	0.198	M323	0.005	G352	-0.032
A36	-0.084	A65	0.097	T324	0.045	K353	0.607
E37	-0.486	G66	-0.038	Q325	-0.081	G354	0.033
D38	-0.589	D67	-0.774	G326	0.040	D355	-0.567
L39	0.027	K68	0.519	F327	0.051	F356	-0.034
F40	-0.008	W69	0.049	W328	2.092	R357	0.719
Y41	-0.020	S70	-0.021	E329	-0.882	I358	0.027
Q42	0.006	A71	0.076	N330	-0.109	L359	-0.048
S43	-0.006	F72	-0.009	S331	-0.079	M360	-0.027
S44	-0.036	L73	-0.001	M332	0.045	C361	0.082
L45	-0.016	K74	0.816	L333	0.021	T362	-0.057
A46	0.048	E75	-0.929	T334	0.093	K363	0.498
S47	-0.049	Q76	-0.006	D335	-0.621	V364	0.026
						T365	-1.006

Table S3: PC^{AA} for ACE2 AAs of OV model. Color coded according to **Figure 13**. The residues in ACE2: 19-88 and 319-365.

AAs	PC^{AA}	AAs	PC^{AA}	AAs	PC^{AA}	AAs	PC^{AA}
S19	0.672	W48	0.028	S77	-0.016	P336	0.050
T20	-0.131	N49	0.027	T78	-0.066	G337	0.006
I21	-0.004	Y50	0.033	L79	-0.057	N338	0.126
E22	-0.399	N51	-0.057	A80	-0.019	V339	0.049
E23	-0.670	T52	-0.063	Q81	0.028	Q340	-0.070
Q24	0.024	N53	-0.003	M82	-0.092	K341	0.718
A25	0.002	I54	0.004	Y83	-0.065	A342	0.041
K26	0.505	T55	-0.066	P84	0.077	V343	-0.084
T27	-0.071	E56	-0.828	L85	0.161	C344	0.136
F28	-0.005	E57	-0.918	Q86	-0.165	H345	-0.127
L29	-0.020	N58	0.080	E87	-0.953	P346	0.094
D30	-0.821	V59	0.006	I88	-1.013	T347	-0.033
K31	0.634	Q60	-0.130	G319	1.026	A348	0.028
F32	-0.074	N61	0.046	L320	-0.146	W349	1.912
N33	-0.072	M62	-0.039	P321	0.135	D350	-0.707
H34	0.161	N63	-0.033	N322	-0.033	L351	-0.052
E35	-0.617	N64	0.239	M323	-0.001	G352	-0.075
A36	-0.050	A65	0.098	T324	0.059	K353	0.664
E37	-0.594	G66	-0.031	Q325	-0.041	G354	-0.048
D38	-0.508	D67	-0.796	G326	0.060	D355	-0.604
L39	-0.022	K68	0.606	F327	0.024	F356	-0.032
F40	-0.010	W69	-0.004	W328	0.101	R357	0.736
Y41	-0.064	S70	-0.027	E329	-0.531	I358	0.033
Q42	0.066	A71	-0.026	N330	-0.075	L359	-0.062
S43	0.027	F72	0.022	S331	-0.072	M360	-0.001
S44	-0.047	L73	0.008	M332	0.033	C361	0.048
L45	-0.009	K74	0.990	L333	0.002	T362	-0.078
A46	0.074	E75	-0.808	T334	0.057	K363	0.401
S47	-0.044	Q76	-0.059	D335	-0.471	V364	0.013
						T365	-0.992

Table S4: PC^{AA} for RBD AAs of WT model. Color coded according to **Figure 13**. The residues in RBD: 333-526.

AAs	PC^{AA}	AAs	PC^{AA}	AAs	PC^{AA}	AAs	PC^{AA}
T333	0.510	V382	-0.022	G431	-0.040	C480	-0.078
N334	-0.090	S383	-0.106	C432	0.079	N481	0.074
L335	0.159	P384	0.171	V433	-0.036	G482	-0.065
C336	-0.193	T385	0.050	I434	-0.014	V483	-0.020
P337	0.195	K386	0.448	A435	-0.033	E484	-0.622
F338	-0.069	L387	-0.038	W436	0.017	G485	-0.074
G339	0.105	N388	-0.010	N437	0.079	F486	0.048
E340	-0.458	D389	-0.482	S438	-0.225	N487	-0.056
V341	-0.063	L390	0.001	N439	0.072	C488	0.064
F342	0.001	C391	0.008	N440	-0.010	Y489	0.003
N343	-0.050	F392	-0.021	L441	-0.008	F490	-0.056
A344	0.007	T393	-0.064	D442	-0.675	P491	0.019
T345	0.036	N394	0.077	S443	0.027	L492	0.066
R346	0.866	V395	0.017	K444	0.613	Q493	0.018
F347	0.006	Y396	-0.173	V445	0.132	S494	-0.043
A348	0.005	A397	0.005	G446	0.052	Y495	-0.213
S349	0.050	D398	-0.647	G447	0.030	G496	0.065
V350	-0.007	S399	0.063	N448	0.037	F497	0.029
Y351	0.064	F400	0.016	Y449	-0.117	Q498	0.008
A352	-0.051	V401	0.024	N450	0.160	P499	0.178
W353	-0.017	I402	-0.048	Y451	-0.101	T500	-0.058
N354	-0.006	R403	0.816	L452	-0.010	N501	-0.118
R355	0.720	G404	0.041	Y453	-0.139	G502	0.020
K356	0.458	D405	-0.702	R454	0.687	V503	0.043
R357	0.914	E406	-0.759	L455	-0.054	G504	0.098
I358	-0.132	V407	-0.034	F456	0.052	Y505	-0.429
S359	-0.046	R408	0.828	R457	0.753	Q506	-0.033
N360	-0.028	Q409	-0.013	K458	0.551	P507	0.140
C361	0.048	I410	0.003	S459	-0.113	Y508	-0.024
V362	-0.112	A411	-0.106	N460	0.047	R509	0.683
A363	0.031	P412	0.144	L461	0.014	V510	0.000
D364	-0.651	G413	-0.040	K462	0.397	V511	-0.076
Y365	-0.093	Q414	-0.014	P463	0.128	V512	0.012
S366	-0.101	T415	0.022	F464	-0.052	L513	-0.007
V367	-0.027	G416	-0.078	E465	-0.433	S514	0.001
L368	-0.032	K417	0.493	R466	0.784	F515	0.093
Y369	0.003	I418	-0.045	D467	-0.598	E516	-0.646
N370	-0.053	A419	0.103	I468	0.037	L517	0.004
S371	-0.100	D420	-0.832	S469	-0.130	L518	-0.031
A372	0.146	Y421	0.031	T470	0.079	H519	0.078
S373	-0.099	N422	-0.034	E471	-0.508	A520	-0.109
F374	0.041	Y423	-0.136	I472	-0.005	P521	0.120
S375	0.019	K424	0.660	Y473	-0.100	A522	0.076
T376	0.038	L425	-0.013	Q474	0.024	T523	-0.112
F377	0.047	P426	0.084	A475	-0.024	V524	-0.044
K378	0.819	D427	-0.628	G476	0.079	C525	0.069
C379	0.027	D428	-0.986	S477	0.015	G526	-0.483
Y380	-0.035	F429	-0.016	T478	-0.125		
G381	0.026	T430	-0.037	P479	0.132		

Table S5: PC^{AA} for RBD AAs of OV model. Color coded according to **Figure 13**. The residues in RBD: 333-526.

AAs	PC^{AA}	AAs	PC^{AA}	AAs	PC^{AA}	AAs	PC^{AA}
T333	0.914	V382	-0.067	G431	-0.046	C480	-0.110
N334	-0.039	S383	-0.019	C432	0.106	N481	0.056
L335	0.093	P384	0.040	V433	-0.062	G482	-0.005
C336	-0.172	T385	-0.006	I434	-0.009	V483	-0.035
P337	0.173	K386	0.513	A435	0.011	A484	0.015
F338	-0.077	L387	0.084	W436	0.057	G485	-0.087
D339	-0.764	N388	-0.150	N437	0.095	F486	0.170
E340	-0.442	D389	-0.923	S438	-0.231	N487	0.030
V341	-0.053	L390	-0.017	N439	0.059	C488	0.031
F342	-0.011	C391	0.000	K440	0.568	Y489	-0.049
N343	-0.130	F392	-0.046	L441	-0.025	F490	0.026
A344	-0.010	T393	-0.011	D442	-0.734	P491	0.159
T345	0.011	N394	0.134	S443	0.032	L492	0.007
R346	0.949	V395	-0.013	K444	0.647	R493	0.721
F347	0.032	Y396	-0.165	V445	0.092	S494	-0.073
A348	0.082	A397	0.019	S446	0.097	Y495	-0.153
S349	0.055	D398	-0.646	G447	-0.066	S496	-0.103
V350	0.002	S399	0.051	N448	0.047	F497	0.030
Y351	0.072	F400	0.010	Y449	-0.165	R498	0.692
A352	-0.067	V401	0.035	N450	0.153	P499	0.112
W353	-0.005	I402	-0.045	Y451	-0.114	T500	-0.058
N354	-0.001	R403	0.728	L452	0.001	Y501	0.032
R355	0.713	G404	0.005	Y453	-0.160	G502	0.026
K356	0.434	D405	-0.757	R454	0.680	V503	0.036
R357	0.795	E406	-0.779	L455	-0.022	G504	0.094
I358	-0.095	V407	-0.009	F456	0.049	H505	-0.012
S359	-0.009	R408	0.786	R457	0.791	Q506	-0.101
N360	0.027	Q409	0.002	K458	0.546	P507	0.141
C361	0.138	I410	0.037	S459	-0.131	Y508	-0.063
V362	-0.080	A411	-0.099	N460	0.005	R509	0.652
A363	-0.073	P412	0.131	L461	0.009	V510	-0.007
D364	-0.526	G413	-0.066	K462	0.402	V511	-0.055
Y365	0.059	Q414	0.071	P463	0.137	V512	-0.005
S366	0.064	T415	-0.014	F464	-0.053	L513	0.001
V367	-0.013	G416	-0.039	E465	-0.463	S514	-0.022
L368	0.036	N417	0.059	R466	0.799	F515	0.094
Y369	-0.096	I418	-0.018	D467	-0.676	E516	-0.692
N370	-0.005	A419	0.073	I468	0.060	L517	0.072
L371	-0.043	D420	-0.811	S469	0.004	L518	-0.039
A372	-0.083	Y421	0.036	T470	0.064	H519	0.069
P373	0.088	N422	-0.043	E471	-0.541	A520	-0.144
F374	0.091	Y423	-0.125	I472	-0.021	P521	0.138
F375	-0.057	K424	0.631	Y473	-0.098	A522	0.025
T376	-0.016	L425	0.013	Q474	0.092	T523	-0.157
F377	0.066	P426	0.138	A475	0.024	V524	-0.040
K378	0.800	D427	-0.558	G476	0.026	C525	0.066
C379	0.050	D428	-0.949	N477	0.117	G526	-0.813
Y380	-0.179	F429	-0.039	K478	0.775		
G381	0.175	T430	-0.048	P479	0.120		

Supplementary References:

1. VASP - Vienna Ab initio Simulation Package. 1 November, 2021]; Available from: https://www.vasp.at/.
2. Ching, W.-Y. and P. Rulis, Electronic Structure Methods for Complex Materials: The orthogonalized linear combination of atomic orbitals. **2012**, Oxford, UK: Oxford University Press.
3. Adhikari, P., R. Khaoulaf, H. Ez-Zahraouy, and W.-Y. Ching, Complex interplay of interatomic bonding in a multi-component pyrophosphate crystal: K2Mg (H2P2O7) 2· 2H2O. *Royal Society open science*, **2017**, 4(12), 170982, DOI: 10.1098/rsos.170982.
4. San, S., N. Li, Y. Tao, W. Zhang, and W.Y. Ching, Understanding the atomic and electronic origin of mechanical property in thaumasite and ettringite mineral crystals. *Journal of the American Ceramic Society*, **2018**, 101(11), 5177-5187, DOI: 10.1111/jace.15774.
5. Zhang, W., Y. Liu, Y. Zhou, W.-Y. Ching, Q. Li, W. Li, J. Yang, and B. Liu, Anti-perovskite carbides and nitrides A3BX: A new family of damage tolerant ceramics. *Journal of Materials Science & Technology*, **2020**, 40, 64-71, DOI: 10.1016/j.jmst.2019.08.043.
6. Adhikari, P., N. Li, M. Shin, N.F. Steinmetz, R. Twarock, R. Podgornik, and W.-Y. Ching, Intra- and intermolecular atomic-scale interactions in the receptor binding domain of SARS-CoV-2 spike protein: implication for ACE2 receptor binding. *Physical Chemistry Chemical Physics*, **2020**, 22(33), 18272-18283, DOI: 10.1039/D0CP03145C.
7. Hasan, S., K. Baral, N. Li, and W.-Y. Ching, Structural and physical properties of 99 complex bulk chalcogenides crystals using first-principles calculations. *Scientific reports*, **2021**, 11(1), 1-18, DOI: 10.1038/s41598-021-89281-6.
8. Shafei, L., P. Adhikari, and W.-Y. Ching, DFT Study of Electronic Structure and Optical Properties of Kaolinite, Muscovite, and Montmorillonite. *Crystals*, **2021**, 11(6), 618, DOI: 10.3390/cryst11060618.
9. Baral, K., S. San, R. Sakidja, A. Couet, K. Sridharan, and W.-Y. Ching, Temperature-Dependent Properties of Molten Li2BeF4 Salt Using Ab Initio Molecular Dynamics. *ACS omega*, **2021**, 6(30), 19822-19835, DOI: 10.1021/acsomega.1c02528.
10. Poudel, L., R. Twarock, N.F. Steinmetz, R. Podgornik, and W.-Y. Ching, Impact of hydrogen bonding in the binding site between capsid protein and MS2 bacteriophage ssRNA. *The Journal of Physical Chemistry B*, **2017**, 121(26), 6321-6330, DOI: 10.1021/acs.jpcb.7b02569.
11. Poudel, L., C. Tamerler, A. Misra, and W.-Y. Ching, Atomic-scale quantification of interfacial binding between peptides and inorganic crystals: The case of calcium carbonate binding peptide on aragonite. *The Journal of Physical Chemistry C*, **2017**, 121(51), 28354-28363, DOI: 10.1021/acs.jpcc.7b10004.
12. Jawad, B., L. Poudel, R. Podgornik, N.F. Steinmetz, and W.-Y. Ching, Molecular mechanism and binding free energy of doxorubicin intercalation in DNA. *Physical Chemistry Chemical Physics*, **2019**, 21(7), 3877-3893, DOI: 10.1039/C8CP06776G.
13. Ching, W.Y., Encyclopedia of materials: technical ceramics and glasses. Ceramic Genome: Total bond order density. **2021**: Elsevier.
14. Jawad, B., L. Poudel, R. Podgornik, and W.-Y. Ching, Thermodynamic Dissection of the Intercalation Binding Process of Doxorubicin to dsDNA with Implications of Ionic and Solvent Effects. *The Journal of Physical Chemistry B*, **2020**, 124(36), 7803-7818, DOI: 10.1021/acs.jpcb.0c05840.
15. Baral, K., A. Li, and W.-Y. Ching, Ab Initio Study of Hydrolysis Effects in Single and Ion-Exchanged Alkali Aluminosilicate Glasses. *The Journal of Physical Chemistry B*, **2020**, 124(38), 8418-8433, DOI: 10.1021/acs.jpcb.0c05875.

16. Yao, M., Z. Shi, P. Zhang, W.-J. Ong, J. Jiang, W.-Y. Ching, and N. Li, Density Functional Theory Study of Single Metal Atoms Embedded into MBene for Electrocatalytic Conversion of N2 to NH3. *ACS Applied Nano Materials*, **2020**, 3(10), 9870-9879, DOI: 10.1021/acsanm.0c01922.
17. Adhikari, P. and W.-Y. Ching, Amino acid interacting network in the receptor-binding domain of SARS-CoV-2 spike protein. *RSC Advances* **2020**, 10, 39831-39841, DOI: 10.1039/d0ra08222h.
18. Ching, W.-Y., P. Adhikari, B. Jawad, and R. Podgornik, Ultra-Large-Scale Ab Initio Quantum Chemical Computation of Bio-Molecular Systems: The Case of Spike Protein of SARS-CoV-2 Virus. . *Computational and Structural Biotechnology Journal* **2021**, 19, 1288-1301, DOI: 10.1016/j.csbj.2021.02.004.
19. Jawad, B., P. Adhikari, R. Podgornik, and W.-Y. Ching, Key interacting residues between RBD of SARS-CoV-2 and ACE2 receptor: Combination of molecular dynamic simulation and density functional calculation. *Journal of Chemical Information and Modeling*, **2021**, 61, 4425-4441, DOI: 10.1021/acs.jcim.1c00560.
20. Adhikari, P., R. Podgornik, B. Jawad, and W.-Y. Ching, First-Principles Simulation of Dielectric Function in Biomolecules. *Materials*, **2021**, 14(19), 5774, DOI: 10.3390/ma14195774.
21. Baral, K., P. Adhikari, B. Jawad, R. Podgornik, and W.-Y. Ching, Solvent Effect on the Structure and Properties of RGD Peptide (1FUV) at Body Temperature (310 K) Using Ab Initio Molecular Dynamics. *Polymers*, **2021**, 13(19), 3434, DOI: 10.3390/polym13193434.
22. Jawad, B., P. Adhikari, K. Cheng, R. Podgornik, and W.-Y. Ching, Computational Design of Miniproteins as SARS-CoV-2 Therapeutic Inhibitors. *International Journal of Molecular Sciences*, **2022**, 23(2), 838, DOI: 10.3390/ijms23020838.
23. Adhikari, P., B. Jawad, P. Rao, R. Podgornik, and W.-Y. Ching, Delta variant with P681R critical mutation revealed by ultra-large atomic-scale ab initio simulation: Implications for the fundamentals of biomolecular interactions. *Viruses*, **2022**, 14(3), 465, DOI: 10.3390/v14030465.
24. Adhikari, P., B. Jawad, R. Podgornik, and W.-Y. Ching, Mutations of Omicron variant at the interface of the receptor domain motif and human angiotensin-converting enzyme-2. *International journal of molecular sciences*, **2022**, 23(5), 2870, DOI: 10.3390/ijms23052870.
25. Jawad, B., P. Adhikari, R. Podgornik, and W.-Y. Ching, Binding Interactions between Receptor-Binding Domain of Spike Protein and Human Angiotensin Converting Enzyme-2 in Omicron Variant. *The journal of physical chemistry letters*, **2022**, 13, 3915-3921, DOI: 10.1021/acs.jpclett.2c00423.
26. Baral, K. and W.-Y. Ching, Electronic structures and physical properties of Na2O doped silicate glass. *Journal of Applied Physics*, **2017**, 121(24), 245103, DOI: 10.1063/1.4987033.
27. Baral, K., A. Li, and W.-Y. Ching, Ab initio modeling of structure and properties of single and mixed alkali silicate glasses. *The Journal of Physical Chemistry A*, **2017**, 121(40), 7697-7708, DOI: 10.1021/acs.jpca.7b06530.
28. Ching, W.Y., M. Yoshiya, P. Adhikari, P. Rulis, Y. Ikuhara, and I. Tanaka, First-principles study in an inter-granular glassy film model of silicon nitride. *Journal of the American Ceramic Society*, **2018**, 101(7), 2673-2688, DOI: 10.1111/jace.15538.
29. Baral, K., A. Li, and W.Y. Ching, Understanding the atomistic origin of hydration effects in single and mixed bulk alkali-silicate glasses. *Journal of the American Ceramic Society*, **2019**, 102(1), 207-221, DOI: 10.1111/jace.15917.
30. Baral, K., A. Li, and W.-Y. Ching, Ab initio molecular dynamics simulation of Na-doped aluminosilicate glasses and glass-water interaction. *AIP Advances*, **2019**, 9(7), 075218, DOI: 10.1063/1.5092617.
31. Poudel, L., R. Podgornik, and W.-Y. Ching, The hydration effect and selectivity of alkali metal ions on poly (ethylene glycol) models in cyclic and linear topology. *The Journal of Physical Chemistry A*, **2017**, 121(24), 4721-4731, DOI: 10.1021/acs.jpca.7b04061.

32. Adhikari, P., N. Li, P. Rulis, and W.-Y. Ching, Deformation behavior of an amorphous zeolitic imidazolate framework–from a supersoft material to a complex organometallic alloy. *Physical Chemistry Chemical Physics*, **2018**, 20(46), 29001-29011, DOI: 10.1039/C8CP05610B.
33. Dharmawardhana, C., A. Misra, and W.-Y. Ching, Theoretical investigation of C-(A)-SH (I) cement hydrates. *Construction and Building Materials*, **2018**, 184, 536-548, DOI: 10.1016/j.conbuildmat.2018.07.004.
34. Tao, Y., W. Zhang, N. Li, W.-Y. Ching, F. Wang, and S. Hu, Atomic-level insights into the influence of zinc incorporation on clinker hydration reactivity. *Open Ceramics*, **2020**, 1, 100004, DOI: 10.1016/j.oceram.2020.100004.
35. San, S. and W.-Y. Ching, Subtle variations of the electronic structure and mechanical properties of high entropy alloys with 50% carbon composites. *Frontiers in Materials*, **2020**, 7, 371, DOI: 10.3389/fmats.2020.575262.
36. San, S., Y. Tong, H. Bei, B. Kombaiah, Y. Zhang, and W.-Y. Ching, First-principles calculation of lattice distortions in four single phase high entropy alloys with experimental validation. *Materials & Design*, **2021**, 209, 110071, DOI: 10.1016/j.matdes.2021.110071.
37. Kresse, G. and D. Joubert, From ultrasoft pseudopotentials to the projector augmented-wave method. *Physical review b*, **1999**, 59(3), 1758, DOI: 10.1103/PhysRevB.59.1758.
38. Kohn, W. and L.J. Sham, Self-consistent equations including exchange and correlation effects. *Physical review*, **1965**, 140(4A), A1133-A1138, DOI: 10.1103/PhysRev.140.A1133.
39. Mulliken, R., Electronic population analysis on LCAO–MO molecular wave functions. II. Overlap populations, bond orders, and covalent bond energies. *The Journal of Chemical Physics*, **1955**, 23(10), 1841-1846, DOI: 10.1063/1.1740589.
40. Mulliken, R.S., Electronic population analysis on LCAO–MO molecular wave functions. I. *The Journal of Chemical Physics*, **1955**, 23(10), 1833-1840, DOI: 10.1063/1.1740588.
41. Pettersen, E.F., T.D. Goddard, C.C. Huang, G.S. Couch, D.M. Greenblatt, E.C. Meng, and T.E. Ferrin, UCSF Chimera—a visualization system for exploratory research and analysis. *Journal of computational chemistry*, **2004**, 25(13), 1605-1612, DOI: 10.1002/jcc.20084.
